# Quantifying What Better-Ear PTA4 Does Not Capture: High-Frequency and Asymmetric Classification Discordance in Two Independent Adult Population Samples from Germany and the United States

**DOI:** 10.3390/audiolres16040111

**Published:** 2026-07-31

**Authors:** Peizheng Wang, Changgeng Mo, Shangqiguo Wang

**Affiliations:** 1 School of Artificial Intelligence, Shenzhen Technology University, Shenzhen 518118, China; wangpeizheng@sztu.edu.cn; 2 Orka Health Limited, Hong Kong SAR, China; 3 Department of Special Education and Counselling, The Education University of Hong Kong, Hong Kong SAR, China; 4 Faculty of Education, The University of Hong Kong, Hong Kong SAR, China

**Keywords:** pure-tone average, PTA4, high-frequency hearing loss, presbyacusis, asymmetric hearing loss, population audiometry, reclassification, NHANES, HÖRSTAT

## Abstract

**Background/Objectives:** The four-frequency better-ear pure-tone average (PTA4; 0.5–4 kHz) is the dominant population definition of hearing impairment but underweights frequencies above 4 kHz and ignores interaural asymmetry. We quantified, in two independent adult samples, the proportion of PTA4-normal adults with elevated high-frequency or asymmetric profiles and whether this has a self-report correlate. **Methods:** Cross-sectional secondary analysis of two public datasets: the German HÖRSTAT/Aalen sample (2008–2012; *N* = 3105) and pooled US NHANES (2011–March 2020; *N* = 10,575; adults ≥ 20 years). The primary outcome, PTA4-discordant high-frequency loss, was defined a priori as better-ear PTA4 ≤ 25 dB HL with a better-ear 6–8 kHz mean (PTA68) > 25 dB HL, reported as the conditional proportion among PTA4-normal adults. Secondary outcomes were interaural asymmetry ≥ 15 dB and self-rated hearing trouble (NHANES AUQ054 ≥ 3). **Results:** Among PTA4-normal adults, 34.3% (95% CI 32.5–36.2) of the German sample and a survey-weighted 28.2% (25.9–30.5) of the NHANES sample had PTA68 > 25 dB HL. Prevalence rose with age (≈three-fold odds per decade; two-fold for male sex; all *p* < 0.001). Of those with PTA68 asymmetry ≥ 15 dB, 75–85% remained PTA4-normal, although the more stringent unilateral-like pattern was rare (1.5–1.7%). Self-reported trouble was elevated in PTA4-discordant mild (OR 2.56) and strong (OR 3.50) groups (OR 1.43 per 10 dB PTA68). **Conclusions:** Although disagreement between the two classifications is arithmetically expected, since they cover different frequencies, its magnitude is substantial and reproducible: roughly a quarter to a third of PTA4-normal adults (28–34%) carry an elevated PTA68, with a graded self-report correlate consistent with early high-frequency presbyacusis, and the same age structure emerges in two samples measured with different equipment and procedures. Because the 25 dB HL criterion is not age-referenced, an increasing share of the discordance at older ages represents expected presbyacusis rather than pathology, so the older-age figures should be read as counts above a fixed public-health threshold. Reporting a high-frequency companion metric (PTA68 or PTA346) alongside PTA4 would make this information visible without displacing the existing system.

## 1. Introduction

The four-frequency pure-tone average (PTA4), computed in the better ear at 0.5, 1, 2, and 4 kHz, is the principal audiometric summary used by the World Health Organization (WHO), the Global Burden of Disease (GBD) study, and most national hearing-health surveillance programmes to classify adult hearing status [1,2]. The WHO 2021 World Report on Hearing retains better-ear PTA4 as the core metric and sets the threshold for mild hearing loss at ≥20 dB HL, with earlier cutoffs (often >25 dB HL) remaining in wide use for cross-study comparisons [1,3].

PTA4 has two well-known structural limitations when used as a population-health classifier. First, PTA4 weights low and mid-frequency thresholds equally with 4 kHz; it therefore underweights frequencies above 4 kHz, which are typically the earliest and most severely affected in age-related and noise-induced hearing loss [4,5]. Second, the better-ear summary discards information about the worse ear, so interaural asymmetry, which can have localising diagnostic value or affect spatial hearing, is not represented [6]. Both limitations are widely acknowledged in the methodological literature but are rarely quantified simultaneously in the same population sample.

Several large population studies have reported high-frequency hearing loss prevalence using alternative averages. Hoffman et al., in successive NHANES reports, used both PTA4 > 25 dB HL and a high-frequency average across 3, 4, and 6 kHz > 25 dB HL [3,7]. von Gablenz and colleagues, in the pooled German HÖRSTAT/Aalen sample, reported prevalence stratified by sex and age and made international comparisons with US and European studies [8]. Humes et al. most recently combined three NHANES cycles (2011–2020) and documented age- and sex-specific hearing thresholds using better-ear PTA4 under both the >25 dB HL and ≥20 dB HL criteria [9]. These reports, however, do not focus on the within-person discordance between better-ear PTA4 classification and the high-frequency or asymmetric audiometric information that PTA4 ignores, nor do they examine whether that audiometric discordance is detectable in functional self-report. In particular, we are not aware of a quantification of the subgroup of adults classified as normal by better-ear PTA4 yet carrying substantial high-frequency or asymmetric deficits, estimated jointly across independent cohorts within a public-health classification framework. The closest prior analysis, by de Gruy et al. in NHANES, showed that high-frequency loss with normal low-frequency hearing accounts for most hearing-loss configurations and argued that pure-tone-average metrics should extend above 4 kHz [10]; the present study differs by taking the within-person discordance between a normal better-ear PTA4 and an elevated PTA68 as the explicit classification endpoint, quantifying it, together with its classification agreement and its interaural-asymmetry counterpart, jointly across two large country-specific adult samples, and linking it to self-reported difficulty within the PTA4-normal stratum.

We therefore quantified, in two independent adult population samples from different countries, the proportion of PTA4-normal adults who nevertheless carry: (i) elevated high-frequency thresholds captured by a PTA68 metric at 6 and 8 kHz, and (ii) substantial interaural asymmetry. We pre-specified that our primary endpoint would be the within-person discordance between the conventional better-ear PTA4 classification and the 6–8 kHz high-frequency summary. We further examined (iii) whether the age- and sex-specific patterns replicate across the two cohorts; (iv) whether the audiometric discordance is accompanied by self-reported hearing trouble, using NHANES self-rated hearing (AUQ054); and (v) summarised the agreement between the two classifications with Cohen’s κ, reported as a descriptor of classification overlap rather than as a test of either metric. Hereafter we refer to the primary endpoint as PTA4-discordant high-frequency hearing loss, to avoid terminological collision with cochlear-synaptopathy-related “hidden hearing loss” in the Liberman/Kujawa sense [11], with which the present audiometric phenomenon is not to be confused.

We hypothesised that (i) a substantial minority of PTA4-normal adults would carry PTA68 > 25 dB HL; (ii) the age-dependent prevalence pattern would be reproducible across the two independent cohorts, with the steepest rise concentrated in a middle-age window; and (iii) the audiometric discordance would correspond to elevated self-reported hearing trouble, with a graded dose–response to PTA68 elevation after adjustment for age and sex.

## 2. Materials and Methods

### 2.1. Data Sources

This was a pre-specified, descriptive, and analytical cross-sectional secondary analysis of two public adult audiometric datasets. The pooled German HÖRSTAT (Oldenburg/Emden, 2010–2012) and Aalen (2008–2009) sample comprised 3105 adults aged 18–97 years with manual air-conduction audiometry at 250, 500, 1000, 2000, 3000, 4000, 6000, and 8000 Hz in both ears, obtained using the HDA200 circumaural headphones and an ascending procedure as described by Holube and von Gablenz [8]. The US National Health and Nutrition Examination Survey (NHANES) is a continuous, nationally representative probability sample of the US civilian non-institutionalised population conducted by the National Center for Health Statistics. Adult air-conduction audiometry at 500, 1000, 2000, 3000, 4000, 6000, and 8000 Hz was available in cycles 2011–2012 (G), 2015–2016 (I), and 2017–March 2020 pre-pandemic (P). NHANES 2013–14 (cycle H) did not include the audiometric examination module and was therefore excluded from the present analysis. Cycles G and I tested ages 20–69 years; cycle P tested ages 70+ years among adults. Combined, these cycles contributed 10,575 adult participants with at least one audiometric examination attempted. Both datasets are publicly available; no additional ethical approval was required for this secondary analysis.

### 2.2. Variables and Harmonisation

We harmonised the two datasets to a common schema of bilateral air-conduction thresholds at 500–8000 Hz (the HÖRSTAT 250 Hz measurement was excluded because NHANES does not measure it). HÖRSTAT missing-value codes 111 (not measured) and 222 (not valid) were recoded to missing. NHANES “no response” (666) and “could not obtain” (888) were likewise recoded to missing in primary analyses. The 1 kHz measurement used AUXU1K1 (first measurement); the AUXU1K2 retest was used only for reliability auditing. NHANES additionally releases retest thresholds at 6 and 8 kHz (AUXR6K, AUXR8K); these were examined for the purpose of a high-frequency boundary check and are reported in Section 3.11. Sex was coded as male/female and age as years. Regular hearing-aid use was harmonised across NHANES cycles as follows: cycles G and I (AUQ146 = 1 “ever worn hearing aid” AND AUQ152 ∈ {1, 2, 3} “worn almost every day/most days/about half the time in past year”); cycle P (AUQ147 = 1 “now use hearing aid/amplifier/implant” AND AUQ153 ∈ {1, 2, 3}). The HÖRSTAT HearAids variable was treated as regular use by design (coded 1 = aided, 2 = unaided). Cycle-specific skip patterns meant HA questions were asked only to adults 20–69 in cycle G, to all adults in cycle I, and to adults 70+ in cycle P; hearing-aid analyses respected these age bands.

Self-rated hearing status in NHANES was captured by AUQ054 on a five-level Likert scale (1 = Excellent, 2 = Good, 3 = A little trouble, 4 = Moderate trouble, 5 = A lot of trouble; a sixth level, “Deaf”, was recoded to 5 for modelling because *n* = 22 across cycles). AUQ054 was administered to all respondents in all three cycles with near-complete response (non-missingness > 99.9%). A primary binary indicator “any hearing trouble” was defined as AUQ054 ≥ 3.

### 2.3. Derived Audiometric Metrics

For each participant and ear, we computed PTA4 as the mean threshold at 500, 1000, 2000, and 4000 Hz and PTA68 as the mean threshold at 6000 and 8000 Hz, using complete-case logic (all constituent frequencies present for that ear). Bilateral validity required both ears to have a computable metric. Better-ear values were the minimum across the two ears. Interaural differences were |ear-R − ear-L| for each metric. PTA68 was chosen as the primary high-frequency summary, rather than the more common HF4 (3, 4, 6, 8 kHz), to eliminate conceptual overlap with the 4 kHz term already included in PTA4; HF4 and a PTA346 summary (3, 4, 6 kHz) aligning with Hoffman et al. were examined as secondary metrics.

### 2.4. Endpoints

The primary endpoint was PTA4-discordant high-frequency hearing loss, defined as better-ear PTA4 ≤ 25 dB HL AND better-ear PTA68 > 25 dB HL. This adopts the conventional > 25 dB HL criterion for a normal PTA4; the stricter WHO 2021 threshold (≤20 dB HL) is examined as a sensitivity analysis (S3), under which participants with a PTA4 of 20–25 dB HL are instead classified as having mild loss rather than as PTA4-normal. Secondary endpoints were: PTA4-defined loss (better-ear PTA4 > 25 dB HL); PTA4 asymmetry (|ΔPTA4| ≥ 15 dB); PTA68 asymmetry (|ΔPTA68| ≥ 15 dB); and a pragmatic unilateral-like pattern (PTA4-worse ≥ 35 dB HL AND PTA4-better ≤ 20 dB HL). For all analyses, the primary analytic population required valid bilateral complete-case metrics in both ears. Interaural asymmetry was computed as an averaged 6–8 kHz interaural difference rather than a single-frequency rule, to reduce the influence of single-frequency measurement variability at 6 and 8 kHz (asymmetry cutoffs of 10 and 20 dB are reported in Supplementary Table S6).

We further categorised participants with valid PTA4 and PTA68 into four mutually exclusive audiometric phenotypes for self-rated hearing analyses: reference (PTA68 ≤ 25 dB HL), PTA4-discordant mild (PTA68 > 25 and ≤40), PTA4-discordant strong (PTA68 > 40), and PTA4-defined loss (PTA4 > 25 dB HL).

### 2.5. Statistical Analysis

Prevalence estimates were reported with 95% Wilson confidence intervals [12] for unweighted HÖRSTAT estimates and logit-transformed, design-adjusted confidence intervals for survey-weighted NHANES estimates (Taylor-linearized variance estimator as implemented in samplics.TaylorEstimator). The NHANES design used masked stratum (SDMVSTRA) and primary sampling unit (SDMVPSU) variables. Combined sampling weights across cycles were constructed from cycle-specific NHANES MEC weights [13]: WTMEC2YR/2 for cycles G and I and WTMECPRP for cycle P [14]. Because the adult audiometric examination was administered to ages 20–69 in cycles G and I and to ages 70+ only in cycle P, the adult age modules are disjoint by cycle. No additional period rescaling of the cycle P weight was applied, because cycle P is the sole source of the 70+ domain; the pooled weighted estimate for all adults is consequently a synthetic combination of a 2011–2016 working-age frame and a 2017–March 2020 frame for ages 70+, and we refer to it as the pooled NHANES analytic sample rather than as a single contemporaneous US adult cross-section. Strata were verified to be non-overlapping across cycles, so SDMVSTRA and SDMVPSU were used directly in the pooled design. As a sensitivity analysis, rescaling all cycles to a common 7.2-year reference period (a procedure appropriate only when cycles contribute overlapping populations) under-weights the cycle-P-sourced 70+ domain and shifts the overall weighted estimate only modestly, from 24.9% to 24.5%; the small magnitude reflects the small population share of the 70+ domain and confirms that this overall-population companion estimate is not sensitive to this choice. PTA4-discordant HF prevalence was stratified by sex and by ten-year age groups (20–29, 30–39, …, 70–79, 80+).

Logistic regression models estimated the association of age (per 10 years) and sex with (1) PTA4-discordant HF restricted to PTA4-normal participants, (2) PTA4 asymmetry, and (3) PTA68 asymmetry. NHANES models were fitted as design-based weighted logistic regressions with first-order Taylor-linearized variance estimation incorporating the survey weights, SDMVSTRA strata, and SDMVPSU primary sampling units (design degrees of freedom = number of PSUs minus number of strata); HÖRSTAT models used standard binomial GLMs. An age × sex interaction was tested for model (1) in both cohorts. Cohen’s κ for PTA4 > 25 vs. PTA68 > 25 binary agreement was computed per cohort [15], with design-weighted computation for NHANES.

In NHANES, a further logistic model predicted “any hearing trouble” (AUQ054 ≥ 3) from audiometric phenotype (four-level categorical), age, and sex, with design-adjusted SEs as above. A secondary continuous dose–response model regressed any-trouble on PTA68 per 10 dB, age per decade, and sex, restricted to PTA4-normal adults.

Seven sensitivity analyses (S1–S7) varied: the HF metric (HF4, S1; PTA346, S2); the classification thresholds (a matched 20 dB HL threshold applied to both the PTA4-normal criterion, PTA4 ≤ 20 dB HL, and the high-frequency cutoff, PTA68 > 20 dB HL, S3; and a 40 dB HL moderate-severe HF cutoff, S4); the handling of NHANES “could not obtain” codes (S5; recoded to 100 dB HL); the handling of HÖRSTAT thresholds exceeding audiometer maximum output (S6; recoded to max rather than max + 5 dB); and the handling of the NHANES no-response code at 6 or 8 kHz (S7; recoded to the audiometer ceiling). Alternative asymmetry cutoffs (10 and 20 dB) were examined separately and are reported in Supplementary Table S6. A forest plot of PTA4-discordant HF prevalence under each specification was produced to visualise robustness.

High-frequency measurement reliability was examined using the NHANES 6 and 8 kHz retest thresholds (AUXR6K, AUXR8K), comparing each retested ear’s initial and repeat value. Because those retests are contributed almost entirely by severely impaired ears, boundary sensitivity was additionally assessed by simulation on the NHANES analytic sample. Independent Gaussian error was added to each constituent frequency at per-frequency standard deviations of 3, 5, and 7.6 dB (the last being the single-measurement standard deviation implied by the observed 6–8 kHz retest difference standard deviation of 10.8 dB under an assumption of independent, equal-variance repeat measurements, 10.8/√2 = 7.6), and ear-specific and better-ear PTA68 were recomputed within each replicate before reapplying the survey weights, with the better-ear designation held at its observed value so that error entered through the two constituent frequencies of that ear; 2000 replicates were drawn per setting under a fixed random seed, and errors were assumed independent across frequencies and ears. A simulation–extrapolation (SIMEX) analysis assuming a per-frequency standard deviation of 5 dB used λ = 0, 0.5, 1.0, 1.5, and 2.0 with 400 replicates per λ and quadratic extrapolation to λ = −1. These are sensitivity scenarios rather than survey estimates and are reported without confidence intervals. Analyses used Python 3.12 (Python Software Foundation, Wilmington, DE, USA) with the pandas, numpy, scipy, statsmodels, samplics, matplotlib and pyreadstat packages (all open-source, obtained from the Python Package Index).

The primary endpoint (PTA4-discordant HF), the secondary asymmetry and unilateral-like endpoints, and the seven sensitivity specifications were finalised before data extraction. The AUQ054 self-rated hearing analyses, the Cohen’s κ agreement analysis, and the age × sex interaction test were added as planned extensions after the primary analyses were completed and cross-cohort reproducibility was observed. None of these analyses were registered on a public platform. Reporting follows the STROBE guideline for cross-sectional studies [16] (the checklist can be found in the Supplementary Material).

## 3. Results

### 3.1. Sample Characteristics (Table 1)

The HÖRSTAT/Aalen sample had a mean age of 54.6 years (SD 17.5; range 18–97); 46.5% were male. The pooled NHANES adult sample had a mean age of 48.5 years (SD 17.4; range 20–80+), reflecting the inclusion of NHANES 2017–2020 cycle P at ages 70+. Mean better-ear PTA4 was 14.2 dB HL in HÖRSTAT and 13.4 dB HL in NHANES overall; mean better-ear PTA68 was 30.1 and 24.9 dB HL, respectively. Valid bilateral PTA4 was available for 100% of HÖRSTAT (3105/3105) and 88.3% of NHANES (9343/10,575); and valid bilateral PTA68 was available for 97.5% and 85.4%, respectively. The 1618 adults overall (HÖRSTAT 78; NHANES 1540) without valid bilateral PTA68 were concentrated in the NHANES 70+ band. Because the conditional analysis requires a valid PTA4 classification, the operative exclusion is the 315 of 9343 NHANES PTA4-valid adults (3.4%) who additionally lacked valid PTA68, concentrated at ages 70+ (14.8% at 70–79 and 38.2% at 80+; ≤1.3% below 70; Supplementary Table S2) and driven by a “no response” code at 6 or 8 kHz (code 666) rather than “could not obtain” (code 888). Assumption-free (Manski) bounds, obtained by setting every missing PTA68 to its most and least favourable value and therefore requiring no missing-data assumption, leave the monotone age gradient intact under every scenario: 42.1–42.3% at 50–59, 65.4–65.9% at 60–69, 73.7–77.6% at 70–79 and 76.2–88.2% at 80+ (Supplementary Table S3). Only the 80+ band is bounded loosely: 90 PTA4-normal adults are eligible in that band, and 14.4% of them lack a valid PTA68, leaving 77 complete cases. This 14.4% is lower than the 38.2% quoted above for all PTA4-valid adults of that age because participants who lose PTA68 disproportionately already meet the PTA4-defined loss criterion and therefore fall outside the PTA4-normal denominator. Estimates specific to the oldest-old band should therefore be read as bounds rather than as point estimates.

Regular hearing-aid use was 6.3% (196/3105) in HÖRSTAT overall and 1.7% (176/10,572) in NHANES overall; the difference reflects age-composition effects and cycle-specific skip patterns (NHANES cycles G/I did not ask 70+ and cycle P did not ask 20–69, so the NHANES figure is dominated by the 20–69 band, where HA use is low).

**Table 1:** Sample characteristics by cohort.

	HÖRSTAT (DE)	NHANES G + I (20–69)	NHANES P (70+)	NHANES Total
*N* (participants)	3105	9082	1493	10,575
Age, mean (SD)	54.6 (17.5)	44.0 (14.3)	76.2 (3.8)	48.5 (17.4)
Male, *n* (%)	1445 (46.5)	4400 (48.4)	763 (51.1)	5163 (48.8)
Regular HA user, *n* (%)	196 (6.3)	86/9079 (0.9)	90/1493 (6.0)	176/10,572 (1.7)
Better-ear PTA4, mean (SD) dB HL	14.2 (14.3)	10.8 (9.8)	30.2 (14.0)	13.4 (12.3)
Better-ear PTA68, mean (SD) dB HL	30.1 (24.4)	22.0 (17.8)	49.4 (17.7)	24.9 (19.7)
Bilateral PTA4 valid, *n* (%)	3105 (100.0)	–	–	9343 (88.3)
Bilateral PTA68 valid, *n* (%)	3027 (97.5)	–	–	9035 (85.4)

Values are n (%) or mean (SD) unless otherwise stated. For NHANES, G + I cycles tested ages 20–69, and cycle *P* tested ages 70+; HA cell denominators reflect cycle-specific skip patterns. PTA4 = mean of 0.5, 1, 2, 4 kHz; PTA68 = mean of 6, 8 kHz. Of the 9035 NHANES adults with a valid bilateral PTA68, 9028 also have a valid bilateral PTA4 and constitute the complete-case analytic sample used in Section 3.2 and Section 3.7.

### 3.2. Participant Flow

**Figure 1:**
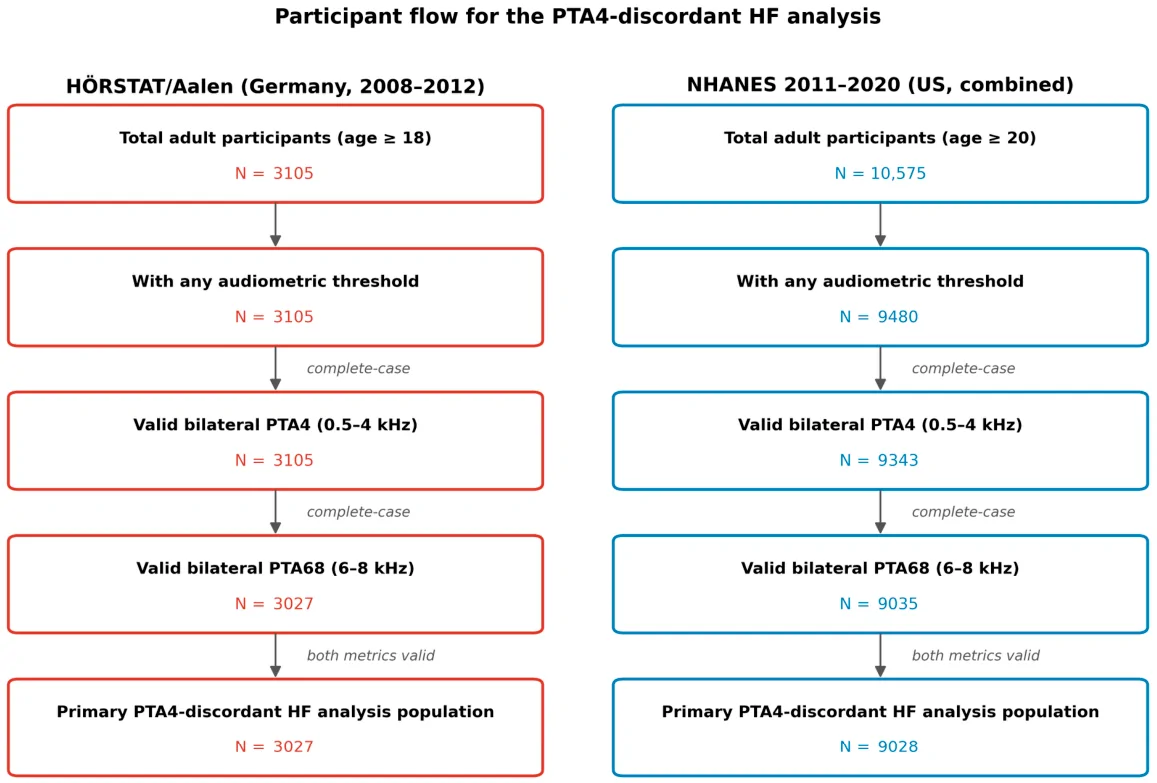
Participant flow for the PTA4-discordant HF analysis, both cohorts.

Figure 1 presents the analytic flow for the PTA4-discordant HF primary endpoint. Complete-case bilateral PTA4 and PTA68 were available for 3027 HÖRSTAT participants (97.5% of the adult sample) and 9028 NHANES adults (85.4%).

### 3.3. Joint Distribution of PTA4 and PTA68 (Figure 2)

**Figure 2:**
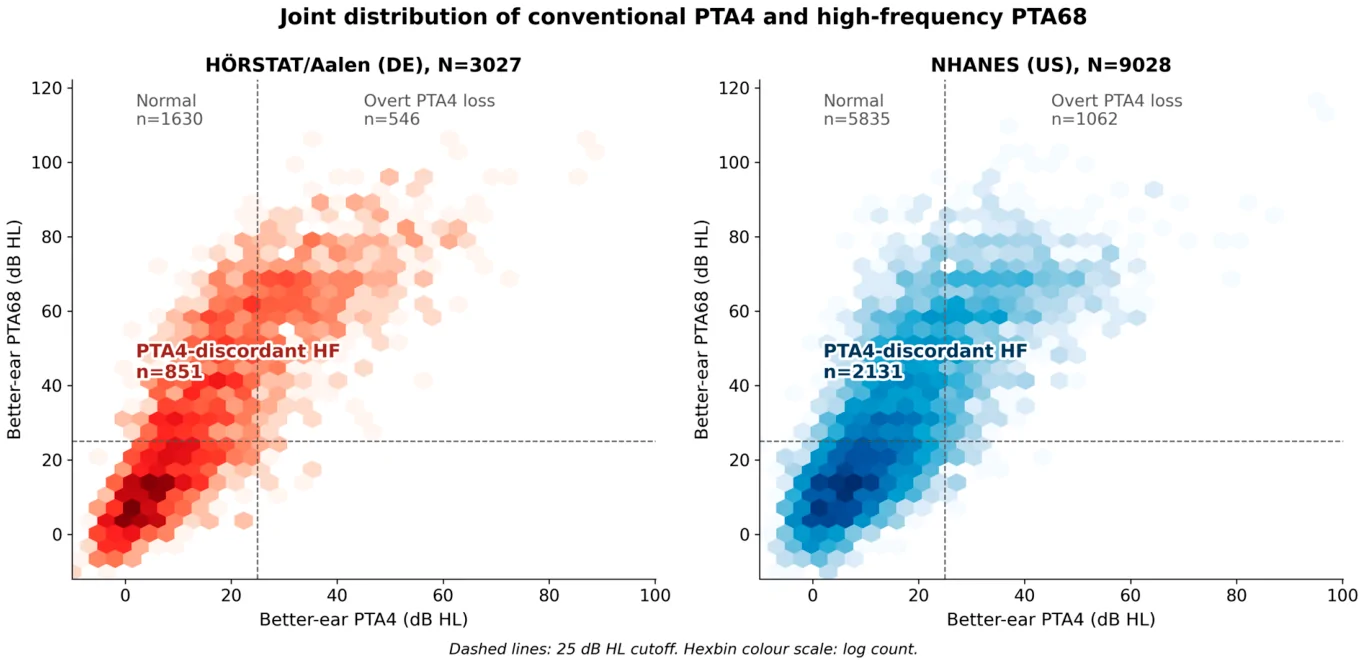
Joint distribution of conventional PTA4 and high-frequency PTA68. Red and blue hexagons denote HÖRSTAT and NHANES, respectively; darker shading indicates larger bin counts on the logarithmic colour scale. Dashed lines indicate the 25 dB HL cutoff.

Figure 2 shows the joint distribution of better-ear PTA4 and PTA68 for both cohorts as a hexagonal density plot. The conventional PTA4 = 25 dB HL decision boundary appears as a vertical reference line. Three structural features are immediately visible. First, the bulk of the density in both cohorts sits to the *left* of the PTA4 boundary (i.e., in the PTA4-normal region), consistent with PTA4 being broadly preserved across adulthood. Second, within this PTA4-normal region the density is highly asymmetric along the PTA68 axis: the upper PTA68 tail extends far beyond the PTA68 = 25 dB HL horizontal reference while the lower tail is sharply bounded at 0 dB HL, so that a substantial density of participants sits in the upper-left quadrant (PTA4 ≤ 25 and PTA68 > 25) where PTA4 classifies as normal but PTA68 is elevated. Third, the lower-right quadrant (PTA4 > 25 and PTA68 ≤ 25) is virtually empty in both cohorts, reflecting the fact that progressive hearing loss tends to affect PTA68 before PTA4 in adult audiometric trajectories. The geometric asymmetry of the joint distribution is the direct visual counterpart of the off-diagonal imbalance of the PTA4 × PTA68 cross-classification described in Section 3.7.

### 3.4. Prevalence of PTA4-Discordant HF Loss

**Figure 3:**
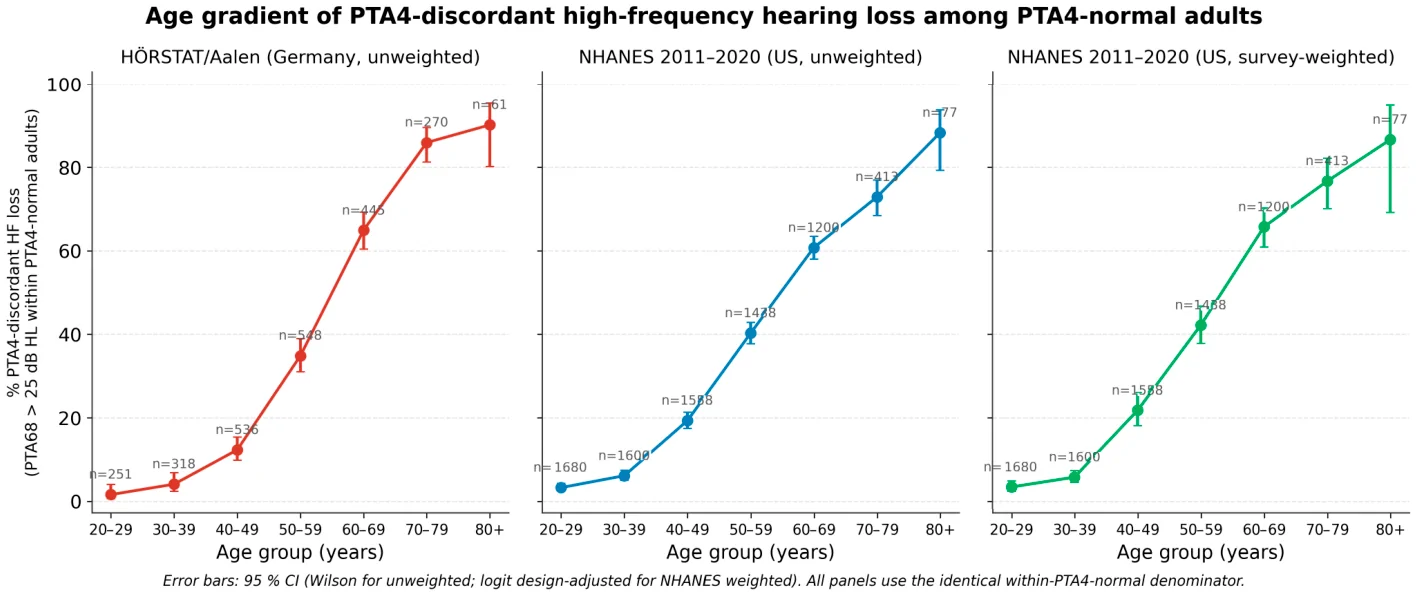
Age gradient of PTA4-discordant HF loss among PTA4-normal adults: HÖRSTAT, NHANES unweighted, and NHANES survey-weighted. All three panels use the identical within-PTA4-normal denominator, yielding closely matched monotone age gradients across cohorts and weighting.

Unweighted PTA4-discordant HF prevalence was 28.1% (95% CI 26.5–29.7) in HÖRSTAT and 23.6% (22.7–24.5) in NHANES (Table 2). The NHANES survey-weighted estimate, computed across all adults with valid bilateral PTA4 and PTA68, was 24.9% (23.0–26.9). Expressed as the primary contrast (the conditional proportion among adults with a normal better-ear PTA4), 34.3% (95% CI 32.5–36.2) of HÖRSTAT and a survey-weighted 28.2% (25.9–30.5) of NHANES PTA4-normal adults carried PTA68 > 25 dB HL; equivalently, of all participants with PTA68 > 25 dB HL, 61% (HÖRSTAT) and 69% (NHANES survey-weighted) retained a normal PTA4 (Supplementary Table S1). A pronounced monotone age gradient within PTA4-normal adults was present in all three views (HÖRSTAT unweighted; NHANES unweighted; NHANES survey-weighted), with the same monotone shape across cohorts and weighting schemes (Figure 3). Among PTA4-normal participants aged 50–59 years, 34.9%, 40.3%, and 42.2% met the PTA4-discordant criterion in the three views, respectively; at 60–69 years, the figures were 64.9%, 60.8%, and 65.7%; at 70–79 years, 85.9%, 72.9%, and 76.7%; and at 80+ years, 90.2%, 88.3%, and 86.6%. The NHANES survey-weighted age gradient therefore reproduces the unweighted NHANES gradient closely (within about five percentage points at every band), whereas absolute prevalence differs between the two cohorts by as much as about ten percentage points in the 40–49 and 70–79 bands; what the cohorts share is the shape of the gradient rather than its level, consistent with the structural, non-cohort-specific nature of the phenomenon.

Male sex was associated with higher PTA4-discordant HF prevalence in both cohorts: 31.6% (men) versus 25.1% (women) in HÖRSTAT, and 25.7% versus 21.6% in NHANES.

### 3.5. Severity Distribution Within PTA4-Normal Participants (Figure 4, Table 3)

Among PTA4-normal participants, PTA68 severity was distributed as follows. In HÖRSTAT: 65.7% PTA68 ≤ 25 dB HL (reference high-frequency category); 17.1% PTA68 26–40 (mild PTA4-discordant, comprising 11.9% at 26–35 and 5.2% at 36–40); and 17.2% PTA68 > 40 (strong PTA4-discordant). NHANES showed a similar structure shifted toward the normal band: 73.2%, 15.8% (12.1% and 3.7%), and 11.0%, respectively; the corresponding survey-weighted values are 71.8%, 15.5%, and 12.6%. The two-band split (26–40; >40) is the one used to define the audiometric phenotypes in Section 2.4, the finer 26–35/36–40 split is descriptive only and is not used elsewhere in the paper. Roughly one in six PTA4-normal HÖRSTAT adults and one in nine PTA4-normal NHANES adults therefore carried a moderate or worse high-frequency deficit (PTA68 > 40 dB HL) not reflected in their better-ear PTA4 classification. We make no inference here about whether this level of high-frequency loss is clinically consequential. Better-ear PTA68 in isolation does not establish hearing-aid candidacy, which depends additionally on speech-frequency thresholds, word-recognition testing, and patient-reported need.

**Figure 4:**
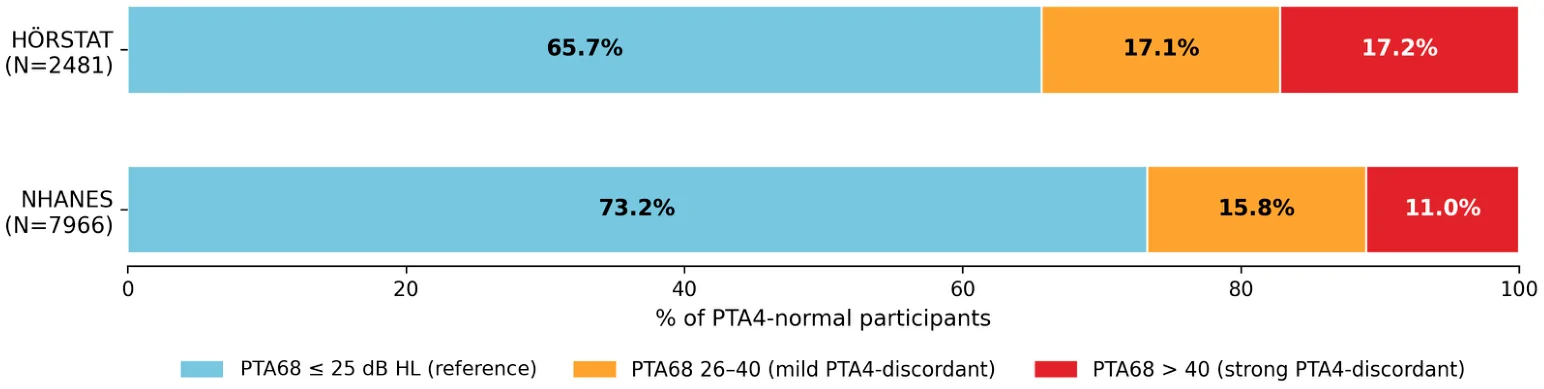
PTA68 severity distribution among PTA4-normal participants.

**Table 3:** PTA68 severity distribution within PTA4-normal participants.

PTA68 Severity Within PTA4-Normal (Unweighted)	HÖRSTAT (*n* = 2481)	NHANES (*n* = 7966)
PTA68 ≤ 25 dB HL (reference high-frequency category)	65.7%	73.2%
PTA68 26–40 dB HL (mild PTA4-discordant)	17.1%	15.8%
of which PTA68 26–35 dB HL	11.9%	12.1%
of which PTA68 36–40 dB HL	5.2%	3.7%
PTA68 > 40 dB HL (strong PTA4-discordant)	17.2%	11.0%

Note. Percentages are unweighted proportions of PTA4-normal participants. The corresponding NHANES survey-weighted values are 71.8%, 15.5% (11.9% and 3.6%), and 12.6%. The 26–40 dB HL band is the mild PTA4-discordant phenotype defined in Section 2.4; the 26–35 and 36–40 sub-bands are descriptive only and are not used elsewhere.

### 3.6. Interaural Asymmetry Among Participants Classified PTA4-Normal (Table 4, Figure 5)

PTA4 asymmetry ≥ 15 dB was present in 5.3% of HÖRSTAT and 5.1% of NHANES participants; PTA68 asymmetry ≥ 15 dB in 16.4% and 16.6%, respectively. Of those with PTA4 asymmetry, 61.0% (HÖRSTAT) and 71.3% (NHANES) still had a better-ear PTA4 ≤ 25 dB HL. Of those with PTA68 asymmetry, 75.2% (HÖRSTAT) and 84.7% (NHANES) remained PTA4-normal. By definition, all unilateral-like participants (1.5% HÖRSTAT; 1.7% NHANES) retained a normal better-ear PTA4. Better-ear PTA4 is not intended as a diagnostic measure of interaural asymmetry, and a better-ear summary discards interaural information by construction; the present analysis therefore quantifies information not represented by the summary rather than demonstrating a failure of PTA4 to perform a function for which it was not designed. The two asymmetry figures reported above also describe different things and should not be conflated. A 15 dB averaged 6–8 kHz interaural difference is common in the general adult population and, given the higher measurement variability at these frequencies, is frequently not clinically consequential; the 16% PTA68 asymmetry figure should therefore not be read as 16% of adults carrying clinically relevant asymmetric loss. The more stringent unilateral-like pattern is the much smaller group at 1.5–1.7%, which a better-ear summary does not represent at all.

**Figure 5:**
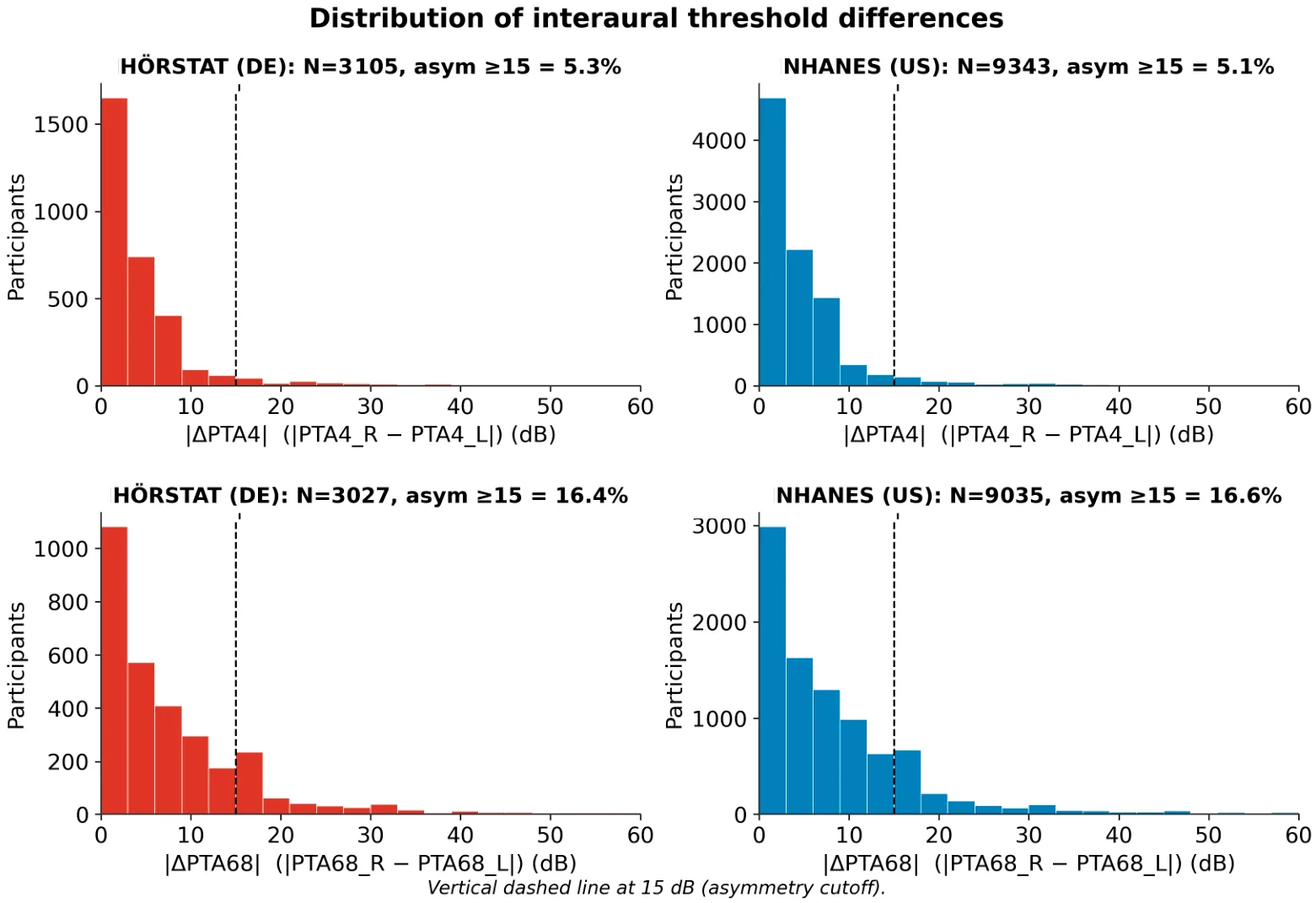
Distribution of interaural threshold differences for PTA4 and PTA68.

**Table 4:** Proportion of participants with interaural asymmetry who remain classified PTA4-normal. Values are unweighted proportions with Wilson 95% confidence intervals in both cohorts.

Condition	Cohort	Cohort Prevalence % (95% CI)	% Still PTA4-Normal (95% CI)
PTA4 asymmetry (Δ ≥ 15 dB)	HÖRSTAT	5.3 (4.5–6.1)	61.0 (53.3–68.1)
PTA4 asymmetry (Δ ≥ 15 dB)	NHANES	5.1 (4.7–5.6)	71.3 (67.1–75.2)
PTA68 asymmetry (Δ ≥ 15 dB)	HÖRSTAT	16.4 (15.1–17.7)	75.2 (71.2–78.8)
PTA68 asymmetry (Δ ≥ 15 dB)	NHANES	16.6 (15.9–17.4)	84.7 (82.8–86.4)
Unilateral-like pattern	HÖRSTAT	1.5 (1.1–2.1)	100.0 (by definition)
Unilateral-like pattern	NHANES	1.7 (1.5–2.0)	100.0 (by definition)

### 3.7. PTA4 vs. PTA68 Classification Agreement (Table 5)

Cohen’s κ for the binary agreement between PTA4 > 25 dB HL and PTA68 > 25 dB HL was 0.40 (95% CI 0.36–0.43) in HÖRSTAT, 0.38 (0.35–0.40) in NHANES unweighted, and 0.36 (0.33–0.39) in NHANES survey-weighted. κ is reported descriptively and is not interpreted as a measure of reliability or comparative validity, because PTA4 and PTA68 summarise different frequency regions and have very different marginal positivity. A low κ is expected by construction and does not indicate that either classifier is unreliable. The substantive observation is the direction of the disagreement rather than its magnitude.

**Table 5:** Cohen’s κ for PTA4 > 25 vs. PTA68 > 25 binary classification agreement.

Cohort	*N*	Cohen’s κ	95% CI
HÖRSTAT (unweighted)	3027	0.398	0.364–0.432
NHANES (unweighted)	9028	0.376	0.353–0.399
NHANES (survey-weighted)	9028	0.358	0.327–0.390

Note. Landis–Koch verbal descriptors are deliberately not applied. κ here compares two classifiers that summarise different frequency regions and have very different marginal positivity, so a low value is expected by construction and is not a measure of the reliability or comparative validity of either metric.

### 3.8. Self-Rated Hearing Within Audiometric Phenotypes (Table 6, Figure 6)

**Figure 6:**
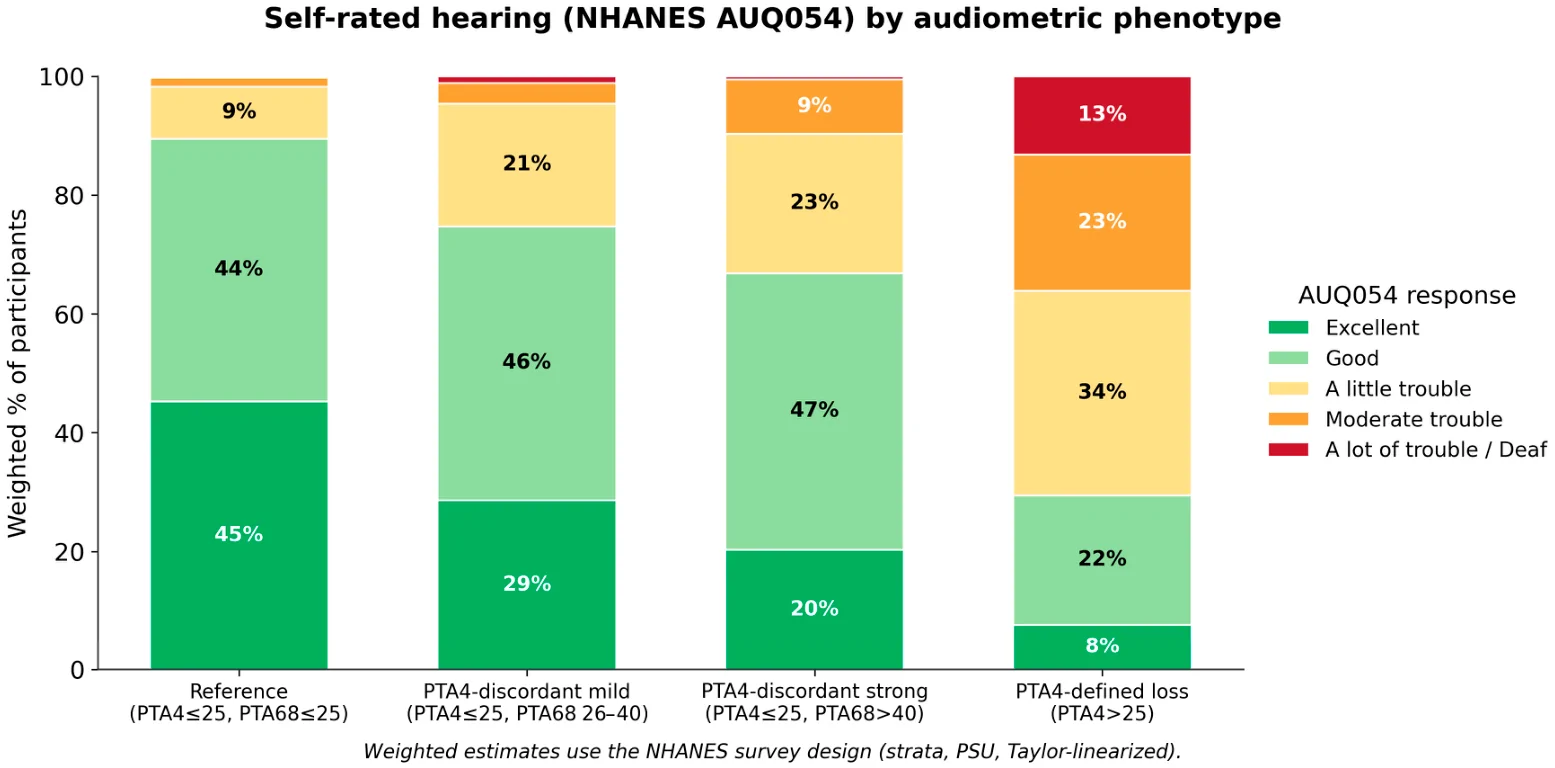
Self-rated hearing distribution across audiometric phenotypes (NHANES survey-weighted).

NHANES self-rated hearing (AUQ054) varied systematically by audiometric phenotype (Figure 6). In the survey-weighted analysis, the proportion reporting any hearing trouble (AUQ054 ≥ 3) was 10.6% in the reference group (PTA4 ≤ 25 and PTA68 ≤ 25), 25.3% in the PTA4-discordant mild group, 33.2% in the PTA4-discordant strong group, and 70.5% in the PTA4-defined loss group. After adjustment for age (per decade) and sex in a design-adjusted logistic model, the odds of any-trouble relative to the reference group were 2.56 (95% CI 1.99–3.29; *p* < 0.001) for PTA4-discordant mild, 3.50 (2.61–4.69; *p* < 0.001) for PTA4-discordant strong, and 16.66 (12.38–22.43; *p* < 0.001) for PTA4-defined loss. A continuous dose–response model restricted to PTA4-normal adults (*n* = 7964) found that each 10 dB worsening of better-ear PTA68 was associated with OR 1.43 (1.34–1.54; *p* < 0.001) for any-trouble, adjusted for age and sex; the age coefficient became non-significant once PTA68 was in the model (OR 1.02, *p* = 0.66). Because age and PTA68 are strongly collinear within this stratum, this attenuation is expected and does not by itself distinguish PTA68 mediating the age association from statistical over-adjustment of two correlated predictors; we report it only as evidence that PTA68 carries most of the self-report-relevant variance that age also indexes, not as a causal or mediation claim. The graded association was materially unchanged after further adjustment for self-reported race/ethnicity and education: the odds of any hearing trouble relative to the reference group were 2.47 (95% CI 1.87–3.26) for PTA4-discordant mild, 3.28 (2.43–4.43) for PTA4-discordant strong, and 15.75 (11.70–21.22) for PTA4-defined loss, and each 10 dB of better-ear PTA68 within PTA4-normal adults retained an odds ratio of 1.41 (1.32–1.51). At the stricter “moderate or worse” threshold (AUQ054 ≥ 4), the phenotype ordering was preserved and steepened (PTA4-discordant mild OR 2.60 [1.40–4.84]; strong 5.47 [3.09–9.69]; PTA4-defined loss 29.0 [18.40–45.72]), indicating that the self-report gradient is not an artifact of the lenient any-trouble threshold. When the three PTA4-normal phenotypes were compared without adjustment within age bands, their ordering was preserved in every band (Supplementary Table S4); the reference-versus-strong contrast was separated in all five bands, whereas the reference-versus-mild contrast was not distinguishable in the two oldest bands.

**Table 6:** NHANES self-rated hearing (AUQ054) by audiometric phenotype (survey-weighted).

Phenotype	*n* (unw)	Any Trouble % (95% CI)	Moderate+ % (95% CI)
Reference (PTA4 ≤ 25, PTA68 ≤ 25)	5834	10.6 (9.5–11.7)	1.8 (1.3–2.4)
PTA4-discordant mild (PTA68 26–40)	1254	25.3 (21.7–29.2)	4.7 (3.2–6.8)
PTA4-discordant strong (PTA68 > 40)	876	33.2 (29.5–37.0)	9.7 (7.5–12.5)
PTA4-defined loss (PTA4 > 25)	1062	70.5 (66.3–74.4)	36.2 (32.1–40.5)

Note: These categories are analytical classification groups defined by the relationship between two summary metrics; they should not be interpreted as distinct clinical entities or diagnostic categories. The three PTA4-normal phenotypes contribute *n* = 7964 participants, two fewer than the 7966 shown in Table 3, because two PTA4-normal participants did not answer the AUQ054 item.

### 3.9. Hearing-Aid Use by Phenotype (Figure 7)

**Figure 7:**
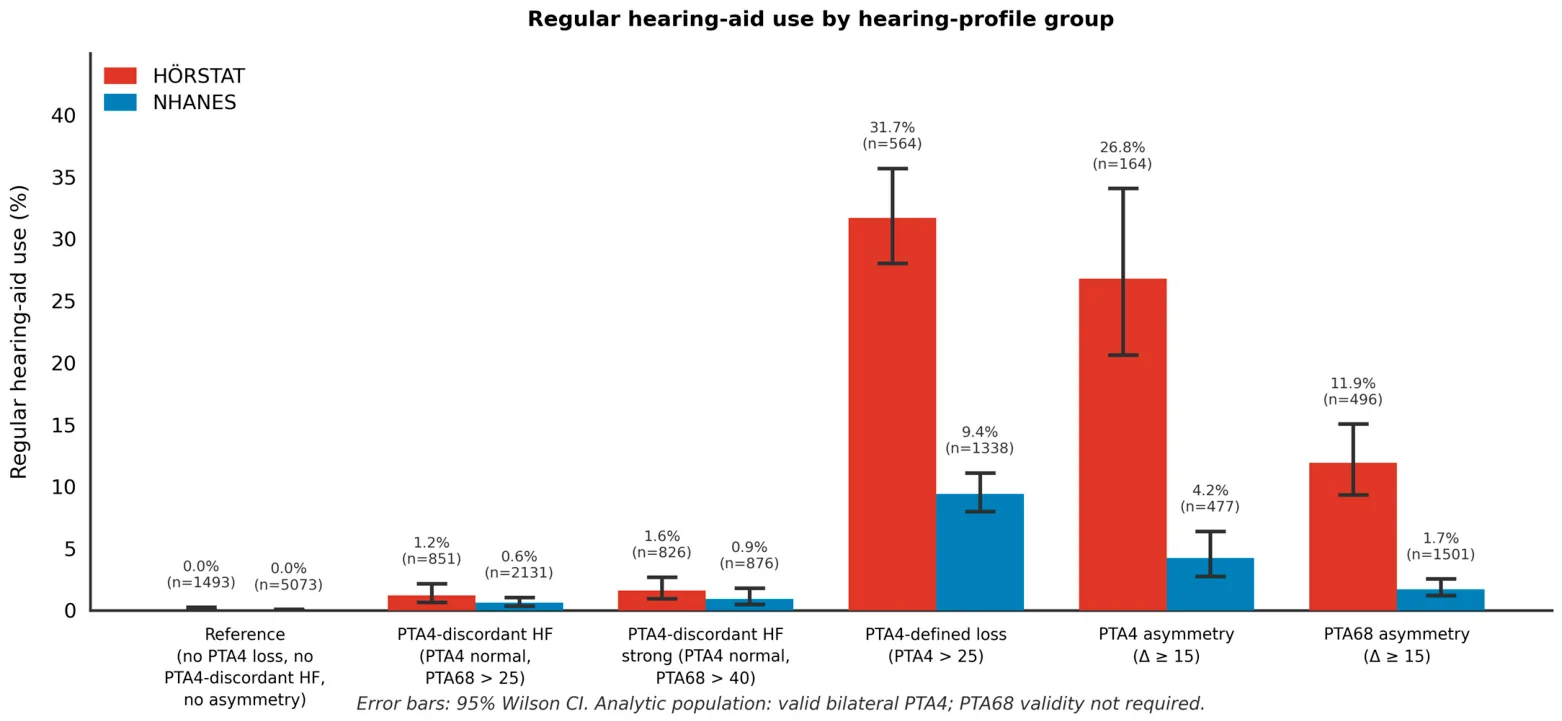
Regular hearing-aid use by hearing-profile group. Analytic population: valid bilateral PTA4 (*N* = 3105 HÖRSTAT/*N* = 9343 NHANES).

Regular hearing-aid use was low in the PTA4-discordant groups (1.2% HÖRSTAT and 0.6% NHANES at the 25 dB HL threshold; 1.6% and 0.9% at 40 dB HL) against 0.0% in the reference group and 31.7% and 9.4% among participants with PTA4-defined loss (Figure 7). Cross-cohort comparison is limited by differing question wording, age coverage, and health-system context, and may reflect national differences in hearing-aid provision rather than audiometric measurement. These figures are descriptive; hearing-aid use is not evidence of clinical appropriateness or of unmet need, and it enters no inferential analysis.

### 3.10. Logistic Regression (Table 7)

In PTA4-normal adults, each 10-year increase in age was associated with odds ratios (OR) for PTA4-discordant HF of 3.71 (95% CI 3.34–4.14; *p* < 0.001) in HÖRSTAT and 2.95 (2.76–3.15; *p* < 0.001) in NHANES (design-adjusted). Male sex was associated with OR 2.14 (1.72–2.68; *p* < 0.001) in HÖRSTAT and OR 2.21 (1.83–2.66; *p* < 0.001) in NHANES. An age × sex interaction term, added to model (1), was non-significant in both cohorts (HÖRSTAT OR 1.04, *p* = 0.73; NHANES OR 1.01, *p* = 0.94), providing no evidence that the age association differed by sex, consistent with parallel age gradients across sex and across cohorts.

**Table 7:** Logistic regression models: predictors of PTA4-discordant HF, asymmetry, and self-reported hearing trouble.

Model/Predictor	Cohort	OR	95% CI	*p*
**Model 1: PTA4-discordant HF|PTA4-normal**				
Age per decade	HÖRSTAT	3.71	3.34–4.14	<0.001
Age per decade	NHANES (design-adj.)	2.95	2.76–3.15	<0.001
Male (vs. female)	HÖRSTAT	2.14	1.72–2.68	<0.001
Male (vs. female)	NHANES (design-adj.)	2.21	1.83–2.66	<0.001
Age × Male (interaction)	HÖRSTAT	1.04	0.84–1.29	0.73
Age × Male (interaction)	NHANES (design-adj.)	1.01	0.89–1.14	0.94
**Model 2: PTA4 asymmetry ≥ 15 dB**				
Age per decade	HÖRSTAT	1.45	1.31–1.61	<0.001
Age per decade	NHANES (design-adj.)	1.35	1.27–1.44	<0.001
Male (vs. female)	HÖRSTAT	1.10	0.80–1.51	0.56
Male (vs. female)	NHANES (design-adj.)	1.64	1.30–2.06	<0.001
**Model 3: PTA68 asymmetry ≥ 15 dB**				
Age per decade	HÖRSTAT	1.38	1.29–1.46	<0.001
Age per decade	NHANES (design-adj.)	1.27	1.21–1.33	<0.001
Male (vs. female)	HÖRSTAT	1.23	1.01–1.49	0.04
Male (vs. female)	NHANES (design-adj.)	1.75	1.49–2.07	<0.001
**Model 4: Any trouble (AUQ054 ≥ 3) ~ phenotype + age + sex (NHANES wt)**				
PTA4-discordant mild (vs. reference)	NHANES (design-adj.)	2.56	1.99–3.29	<0.001
PTA4-discordant strong (vs. reference)	NHANES (design-adj.)	3.50	2.61–4.69	<0.001
PTA4-defined loss (vs. reference)	NHANES (design-adj.)	16.66	12.38–22.43	<0.001
Age per decade	NHANES (design-adj.)	1.07	1.00–1.15	0.05
Male (vs. female)	NHANES (design-adj.)	1.24	1.06–1.45	0.008
**Model 5: Continuous dose–response (PTA4-normal, any-trouble)**				
Better-ear PTA68 (per +10 dB)	NHANES (design-adj.)	1.43	1.34–1.54	<0.001
Age per decade	NHANES (design-adj.)	1.02	0.94–1.10	0.66
Male (vs. female)	NHANES (design-adj.)	1.20	1.01–1.43	0.04

NHANES models are design-based weighted logistic regressions with Taylor-linearized variance (weights, SDMVSTRA strata, SDMVPSU clusters); HÖRSTAT models use standard binomial GLM. Age × sex interaction added to Model 1 as an exploratory planned extension after completion of the primary analyses. Bold rows are model headings and contain no estimates.

For PTA4 asymmetry, the age-decade OR was 1.45 (HÖRSTAT) and 1.35 (NHANES, design-adjusted); for PTA68 asymmetry, 1.38 and 1.27. Male sex carried OR 1.10 (NS; HÖRSTAT) and 1.64 (*p* < 0.001; NHANES) for PTA4 asymmetry and OR 1.23 (*p* = 0.04) and 1.75 (*p* < 0.001) for PTA68 asymmetry. The age-decade OR for PTA4-discordant HF was more than twice that for either asymmetry outcome, consistent with PTA4-discordant HF being driven primarily by progressive high-frequency presbyacusis.

### 3.11. High-Frequency Measurement Reliability and Boundary Sensitivity

Thresholds at 6 and 8 kHz are less repeatable than mid-frequency thresholds and are sensitive to transducer type and placement, so we examined how far the primary classification depends on measurement error near the 25 dB HL boundary. NHANES releases retest thresholds at 6 and 8 kHz (AUXR6K, AUXR8K). Among adults aged 20 years and over, 280 ear-frequency pairs had both an initial and a retest value, contributed by 207 participants. These retests are not a random quality-control sample: the median initial threshold in the retested ears was 75 dB HL (interquartile range 65–85), only 2 of 280 observations were at or below 30 dB HL, and 67.9% were at or above 70 dB HL, consistent with retesting being triggered by absent or unreliable responses in severely impaired ears. Restricting to ears with a complete retested PTA68 (*n* = 73, mean 79.7 dB HL), no ear crossed the 25 dB HL boundary in either direction. The available retest data therefore cannot inform boundary misclassification near 25 dB HL, and the mean retest-minus-initial difference across the 280 ear-frequency pairs (−7.6 dB, SD 10.8) is a property of that severely impaired subgroup and should not be read as a population-level measurement bias.

Boundary sensitivity was therefore assessed by simulation. Within PTA4-normal adults, 23.2% had a better-ear PTA68 within ±5 dB of the 25 dB HL boundary and 14.5% within ±3 dB. Adding independent Gaussian measurement error to each constituent frequency raised the survey-weighted prevalence of PTA4-discordant HF from 28.2% to 30.2%, 30.8%, and 31.9% at per-frequency standard deviations of 3, 5, and 7.6 dB, respectively, with the increase arising because the density of the PTA68 distribution is higher immediately below the boundary than immediately above it. A simulation–extrapolation (SIMEX) analysis assuming a per-frequency standard deviation of 5 dB estimated an error-free prevalence of 23.8%. The two analyses answer different questions and move in opposite directions by construction: adding error asks how the estimate would behave if measurement were noisier than it is, whereas SIMEX extrapolates to the estimate that would be obtained if the constituent thresholds were measured without error. Classical measurement error of plausible magnitude therefore places the primary estimate in the region of 24–31% (Supplementary Table S5). Because the simulation assumes errors independent across frequencies, whereas part of the error at 6 and 8 kHz arises from transducer placement and is therefore correlated within an ear, these scenarios understate the error variance of a two-frequency mean; the 7.6 dB setting should accordingly be read as a conservative upper scenario rather than as a best estimate.

### 3.12. Sensitivity Analyses (Figure 8, Table 8)

**Figure 8:**
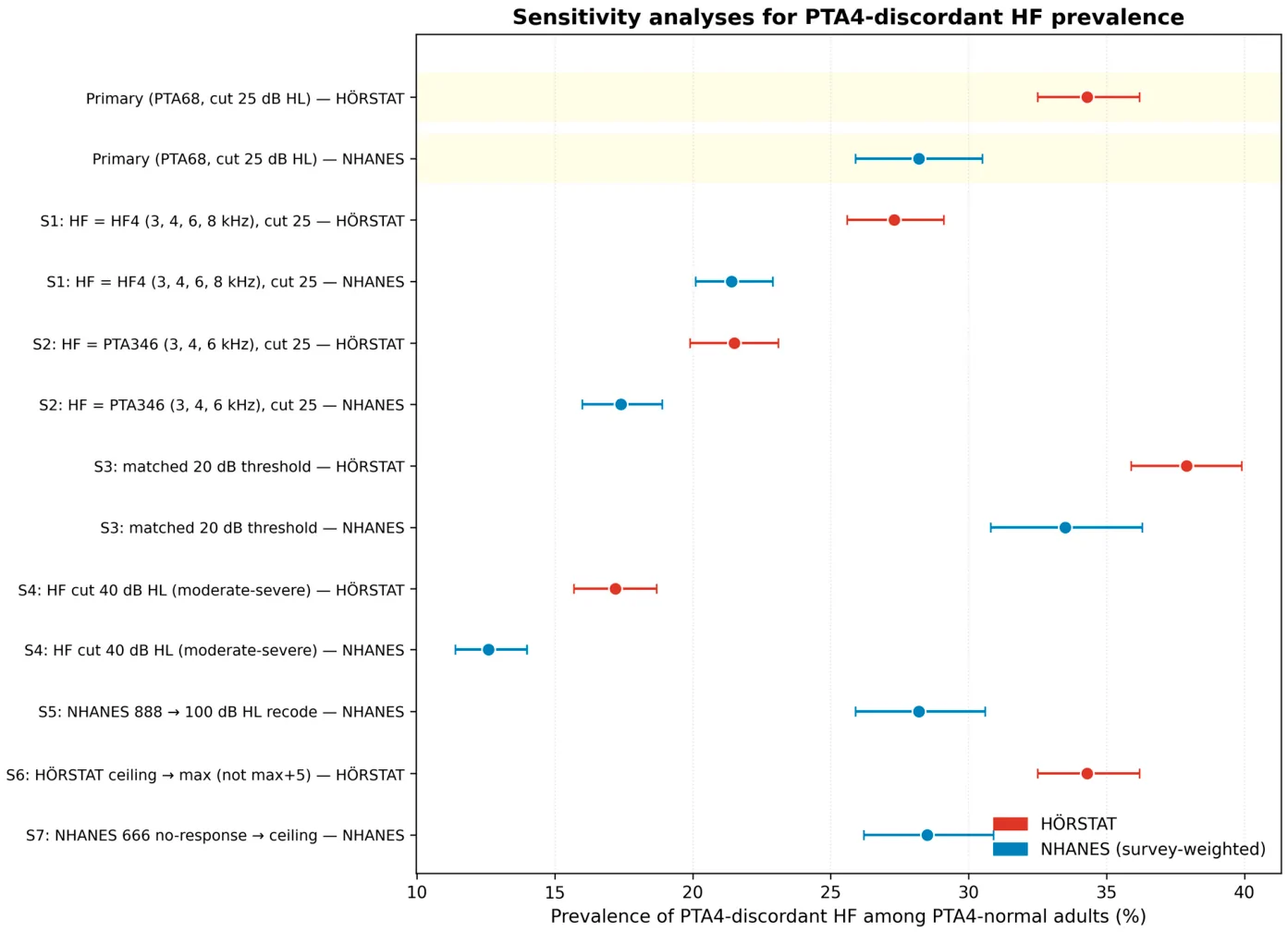
Forest plot of PTA4-discordant HF prevalence, reported on the primary conditional estimand (PTA68 above the stated cutoff among PTA4-normal adults; NHANES survey-weighted), across the primary definition and the seven sensitivity specifications S1–S7 reported in Table 8. Red denotes HÖRSTAT, blue denotes survey-weighted NHANES, and pale-yellow shading identifies the primary estimates. Points and horizontal bars show prevalence estimates and 95% confidence intervals.

PTA4-discordant HF prevalence estimates varied systematically with the choice of HF metric and cutoff but retained cross-cohort consistency (Figure 8); all specifications are reported on the primary conditional estimand (Table 8). Using HF4 (3, 4, 6, 8 kHz) yielded lower prevalence (27.3% HÖRSTAT, 21.4% NHANES) because of the preserved 4 kHz information common to both PTA4 and HF4. Using PTA346 (3, 4, 6 kHz; aligning with Hoffman et al.) yielded the lowest figures (21.5% HÖRSTAT, 17.4% NHANES). Applying a matched 20 dB HL threshold to both the PTA4-normal criterion (PTA4 ≤ 20 dB HL) and the high-frequency cutoff (PTA68 > 20 dB HL) gave 37.9% HÖRSTAT and 33.5% NHANES, confirming that the discordance persists under a stricter definition of normal and is not an artifact of the 20–25 dB HL borderline stratum. At a strict 40 dB HL cutoff, prevalence was 17.2% (HÖRSTAT) and 12.6% (NHANES). Recoding HÖRSTAT ceiling values to the audiometer maximum (S6) left the estimate unchanged at 34.3%. Recoding NHANES “could not obtain” (code 888) values to 100 dB HL (S5) also moved the estimate negligibly (28.2%), but this is because the elevated PTA68 missingness in the older cycle is driven predominantly by the “no response” code (666; 14.3% at 70–79 and 36.9% at 80+ among PTA4-valid adults) rather than by “could not obtain” at 6 or 8 kHz (888; 0.4% and 1.4%), so the 888 recode addresses only a small fraction of the dropout (age-stratified missingness, Supplementary Material). We therefore bounded the conditional discordance without any missing-data assumption: setting every missing PTA68 to its most and least favourable value, the within-PTA4-normal discordance remained 42.1–42.3% at 50–59, 65.4–65.9% at 60–69, 73.7–77.6% at 70–79, and 76.2–88.2% at 80+, so the monotone age gradient held under all scenarios. A complementary point estimate that instead recodes the NHANES no-response code at 6 or 8 kHz to the audiometer ceiling (S7) recovered the older-adult participants excluded by complete-case logic and placed the within-PTA4-normal discordance at 77.5% (70–79) and 88.1% (80+), at the upper edge of these assumption-free bounds and confirming that complete-case exclusion did not inflate the estimate. These bounds are tight because 86–92% of older participants who lose PTA68 already have PTA4-defined loss and so fall outside the PTA4-normal denominator.

**Table 8:** Sensitivity analyses for PTA4-discordant HF prevalence across alternative specifications, reported on the primary conditional estimand (PTA68 > 25 dB HL among PTA4-normal adults; NHANES survey-weighted).

Scenario	HÖRSTAT % (95% CI)	NHANES % (95% CI)
Primary (PTA68, cut 25 dB HL)	34.3 (32.5–36.2)	28.2 (25.9–30.5)
S1: HF = HF4 (3, 4, 6, 8 kHz), cut 25	27.3 (25.6–29.1)	21.4 (20.1–22.9)
S2: HF = PTA346 (3, 4, 6 kHz), cut 25	21.5 (19.9–23.1)	17.4 (16.0–18.9)
S3: matched 20 dB threshold (PTA4 ≤ 20 & PTA68 > 20 dB HL)	37.9 (35.9–39.9)	33.5 (30.8–36.3)
S4: HF cut 40 dB HL (moderate-severe)	17.2 (15.7–18.7)	12.6 (11.4–14.0)
S5: NHANES 888 → 100 dB HL recode	–	28.2 (25.9–30.6)
S6: HÖRSTAT ceiling → max (not max + 5)	34.3 (32.5–36.2)	–
S7: NHANES 666 no-response → ceiling	–	28.5 (26.2–30.9)

## 4. Discussion

Because PTA4 excludes 6 and 8 kHz, the existence and direction of PTA4–PTA68 classification discordance are expected rather than novel. The contribution of the present study is to quantify its magnitude, its age and sex distribution, its stability across alternative operational definitions, its consistency across two adult samples, and its association with a single self-reported hearing-trouble item. Roughly a quarter to a third of PTA4-normal adults in each cohort carried a better-ear PTA68 above 25 dB HL (Table 2, Supplementary Table S1), and the age signature was reproducible across both samples, rising from low single-digit percentages in the third decade of life to ≥60% by age 60–69 in both cohorts, with the steepest rise between 50 and 70 years. The age-decade odds ratio of approximately 3 for PTA4-discordant HF within PTA4-normal participants suggests that the discordance is predominantly a structural consequence of early high-frequency presbyacusis [17], which precedes the rise of PTA4 above the 25 dB HL threshold by a substantial margin.

PTA68 covers the frequency region where age-related and noise-related loss appears earliest, which is why the disagreement runs almost entirely in one direction. Readers should not take the discordance itself as evidence that PTA4 is deficient for the purpose for which it was adopted, which is the summary of speech-frequency hearing; the question raised here is narrower, namely what information a PTA4-only surveillance system discards and how much of it there is.

Three features of the finding are worth emphasising. First, the closely parallel shape of the age trajectories in the two cohorts (Figure 3), despite different countries, sampling frames, audiometric equipment (HDA200 vs. TDH-49/insert), and testing procedures (manual ascending vs. automated modified Hughson-Westlake), supports an interpretation that the PTA4-discordant gap reflects a structural property of the PTA4 metric rather than a cohort artefact; because transducer-related differences are larger at 6 and 8 kHz than at any other audiometric frequency, the recovery of the same age trajectory under two different transducer and procedure combinations argues against a purely measurement-driven or cohort-specific explanation, although it does not eliminate transducer- or procedure-related differences (Section 3.11); the age × sex interaction null (*p* > 0.7 in both cohorts) is consistent with a similar age association in men and women. Second, the effect is not abolished by plausible alternative HF definitions or cutoffs (Figure 8), including the Hoffman-style PTA346 that aligns the HF summary with earlier NHANES reports. Third, the asymmetry analysis (Table 4) shows that the better-ear summary does not represent the majority of participants with substantial interaural differences, including the whole of the pre-specified unilateral-like group, so that asymmetric profiles are not visible in PTA4-based population surveillance. Where the two classifiers disagree, the disagreement is almost entirely in the direction of PTA68 flagging people whom PTA4 does not (Section 3.7).

### 4.1. Functional Relevance: Self-Rated Hearing

The AUQ054 analysis in NHANES indicates that the audiometric discordance has a self-report correlate. After adjustment for age and sex, participants in the PTA4-discordant mild group had more than 2.5-fold odds of reporting any hearing trouble relative to the reference group, and those in the PTA4-discordant strong group had 3.5-fold odds. The continuous dose–response of OR 1.43 per 10 dB of PTA68, with age rendered non-significant once PTA68 entered the model (a pattern consistent with, but given age–PTA68 collinearity not diagnostic of, mediation), is consistent with an association between self-reported difficulty and the high-frequency threshold elevation that PTA4 classification ignores, although the cross-sectional design cannot establish direction. This self-report signal does not substitute for the speech-in-noise [18] or QoL outcomes that would be needed to establish unmet clinical need, but the phenotype ordering is preserved within every age band examined, with the reference-versus-strong contrast separated in all five bands (Supplementary Table S4), so age alone does not fully explain the pattern, although residual confounding within these broad bands cannot be excluded. The magnitude should be read against the reference group rather than against PTA4-defined loss, whose much larger odds ratio simply reflects more hearing loss: relative to the reference group, the absolute excess in any reported trouble is about 15 percentage points in the mild and 23 percentage points in the strong PTA4-discordant group. Self-reported difficulty among adults whose audiograms are considered normal has been described previously [19], and the present analysis locates part of that difficulty in the high-frequency thresholds that a PTA4 summary does not represent.

### 4.2. Sex Differences

Male sex was associated with approximately two-fold odds of PTA4-discordant HF in both cohorts (HÖRSTAT OR 2.14, NHANES design-adjusted OR 2.21), with the age × sex interaction non-significant in each. The near-identical male-to-female odds ratio across two countries, two sampling frames, and two testing procedures indicates that the association is not a cohort-specific artefact. Several lines of evidence point toward differential lifetime exposure histories as the dominant driver: men are more heavily represented in noise-exposed occupations, in which hearing loss is highly prevalent [20], and have historically higher rates of impulsive-noise recreational exposure. A biological contribution has also been proposed, most prominently an oestrogen-mediated cochlear protection hypothesis in premenopausal women [21], although the literature remains mixed. Because our datasets lack harmonised noise histories, we cannot attribute the male excess to any mechanism; we report only that the association is reproducible across cohorts and parallel to the age gradient. The PTA68 asymmetry model showed a larger male effect in NHANES (OR 1.75) than in HÖRSTAT (OR 1.23); we do not have data to explain this difference.

### 4.3. Consistency of the Age Gradient Across Weighting Schemes

Survey-weighted and unweighted NHANES estimates agree closely across all age bands when the within-PTA4-normal denominator used throughout this study is retained: the unweighted NHANES trajectory lies within about five percentage points of the weighted trajectory at every age band, and the two cohorts show the same monotone age pattern although absolute prevalence differs by as much as about ten percentage points in the middle-age bands (Figure 3, Table 2). Sampling-weight artefact is therefore not an explanation for the age gradient. If the denominator is instead expanded to *all* adults with valid metrics in a given age band, the weighted trajectory becomes non-monotone and declines above age 70, because most adults in the oldest bands have already transitioned out of the PTA4-normal pool into PTA4-defined loss rather than because the underlying discordance declines. Because our primary endpoint concerns the discordance *within* PTA4-normal adults, the within-PTA4-normal denominator is used throughout.

### 4.4. Relation to Previous Work

Our findings are consistent with but extend earlier reports. Hoffman et al. reported NHANES high-frequency loss prevalence using PTA346, and von Gablenz et al. reported HÖRSTAT high-frequency loss prevalence and international comparisons [3,7,8]. Neither took the within-person discordance as its principal endpoint. In NHANES, de Gruy et al. compared four pure-tone averages against self-reported difficulty, found that high-frequency loss with normal low-frequency hearing represented roughly 70% of hearing-loss configurations, and concluded that a pure-tone-average metric should incorporate frequencies above 4 kHz [10]. We converge on that recommendation but differ in design, taking the discordance itself as the classification endpoint and extending the same logic to interaural asymmetry. Humes and Zapala recently argued that a single PTA-based severity grade, even when cutoffs are modernised (as in WHO 2021’s ≥ 20 dB HL), sacrifices audiogram configuration information [5]. Our results quantify the scale of that sacrifice in two large adult samples and show that it is dominated by a predictable age window between 50 and 70 years. The HUNT study previously reported population-scale asymmetric hearing loss in adults [6]; our asymmetry-masking analysis shows that most such cases are not captured by a better-ear summary [22]. A national NHANES analysis likewise found asymmetry more than three times as prevalent across 4–8 kHz (9.5%) as across 0.5–4 kHz (2.8%) [23], external corroboration that a better-ear speech-frequency summary omits most high-frequency asymmetry.

The audiometric phenomenon characterised here should not be confused with the cochlear-synaptopathy concept of “hidden hearing loss” developed by Kujawa and Liberman and colleagues [11]. Synaptopathic hearing loss refers to suprathreshold loss of inner-hair-cell synapses and auditory-nerve fibres in the context of normal audiometric thresholds, producing deficits in speech-in-noise performance that are undetected by standard pure-tone audiometry at any frequency [24]. The PTA4-discordant HF phenotype described in the present study is, by contrast, explicitly audiometric: the high-frequency thresholds are elevated and measurable in a standard clinical audiogram, but are hidden only from the PTA4 summary statistic. The two phenomena likely coexist in many adults, differ in their required instrumentation (standard audiometry vs. word-in-noise or electrophysiological tests), and call for different interventions. Our choice of terminology is intended to make this separation explicit.

### 4.5. Public-Health and Policy Implications

These results speak most directly to the choice of audiometric classifier in population-health surveillance, not to clinical decision-making at the individual level. The WHO 2021 World Report on Hearing and GBD 2019 hearing estimates both use better-ear PTA4 as the central metric [1,2]. Such classifiers do not represent high-frequency or asymmetric hearing loss, and at the population level the resulting gap between PTA4 and PTA68 classification is widest in the 50–69 year age window, because most older adults have already moved into PTA4-defined loss. Reporting a PTA68 or PTA346 companion metric alongside PTA4 would make this information visible without changing the familiar PTA4 baseline. We emphasise what the present data do not establish: we did not test whether adding a high-frequency metric improves surveillance accuracy, changes treatment or prognosis, or is justified on cost grounds, and none of these follows from the reclassification counts reported here.

### 4.6. Clinical Implications

This is a surveillance study, and the following observation is offered as a hypothesis for clinical research rather than as validated clinical guidance. The PTA68 measurement is already collected in standard adult audiometry, and within the NHANES PTA4-normal stratum, a higher PTA68 was accompanied by more self-reported difficulty (25% and 33% in the mild and strong PTA4-discordant groups against 11% in the reference group). A normal PTA4 therefore does not by itself exclude measurable high-frequency loss. We make no claim that these patients are underserved, that they would benefit from any intervention, or that PTA68 has established clinical utility: the only functional measure available here is a single self-rating in cross-sectional data, with no speech-in-noise, word-recognition or quality-of-life outcome. An elevated PTA68 in isolation is not an indication for fitting, and delivering verifiable gain at 6 and 8 kHz is constrained by current hearing-aid bandwidth.

### 4.7. Limitations

Eight limitations must be acknowledged. First, speech-in-noise, self-reported functional difficulty, and hearing-related quality-of-life measures remain incomplete in the public releases: NHANES provides only the single AUQ054 self-rating and no speech-in-noise testing, and HÖRSTAT provides neither. The present study partially addresses this gap by reporting a design-adjusted graded relationship between audiometric phenotype and NHANES self-rated hearing trouble, but a formal demonstration of unmet clinical need would require speech-in-noise audiometry (e.g., Digits-in-Noise [25]) and hearing-related QoL instruments not available in either dataset. “PTA4-discordant HF” therefore remains, in strict terms, an audiometric classification discordance with a self-report correlate. Second, PTA68 became progressively incomplete with age in NHANES (for the conditional analysis, missing in 14.8% of PTA4-valid adults aged 70–79 and 38.2% of those aged 80+), driven mainly by the “no response” code at 6 or 8 kHz rather than the “could not obtain” code that sensitivity analysis S5 recodes; complete-case analysis therefore removes severe high-frequency cases from the older bands and could in principle bias the valid-PTA68 denominator toward well-preserved hearing. Two observations limit the impact on the primary endpoint: 86–92% of older participants who lose PTA68 already have PTA4-defined loss and so fall outside the PTA4-normal denominator, and assumption-free bounds on the conditional discordance (Results) leave the monotone age gradient intact under all missing-data scenarios, although the 80+ band (*n* = 90 PTA4-normal) is bounded only loosely (76.2–88.2%). Third, equipment and procedure differ between cohorts (HDA200/manual vs. TDH-49/automated), which at 6 and 8 kHz can produce systematic offsets of a few decibels; for this reason, we compared prevalence at common cutoffs rather than pooling raw thresholds, and our conclusions concern within-cohort patterns and their reproducibility, not pooled estimates. Fourth, the HÖRSTAT public release contains no sampling weights, so HÖRSTAT estimates describe the released sample rather than the German adult population; we relied on the NHANES survey-weighted estimates (Table 2) for nationally representative comparisons. Fifth, the hearing-aid variable had cycle-specific skip patterns in NHANES; we harmonised to a regular-use definition but acknowledge that HA use here functions as a descriptive marker, not as evidence of clinical appropriateness or unmet need. Sixth, we did not stratify results by race or ethnicity. NHANES records participant-reported race/ethnicity (RIDRETH1), and the expected age and sex gradients could interact with lifetime noise and healthcare access differentials across groups; the HÖRSTAT release contains no analogous variable, which would have precluded cross-cohort stratified comparison even if we had pursued this. Beyond the covariate adjustment for race/ethnicity and education reported here, a fully race/ethnicity–stratified analysis remains a planned extension. Seventh, thresholds at 6 and 8 kHz have poorer test–retest reliability than mid-frequency thresholds and are sensitive to transducer type and placement, and a hard 25 dB HL boundary will therefore misclassify some participants in both directions; Section 3.11 quantifies the size of this effect but cannot eliminate it. Eighth, and most fundamentally, the 25 dB HL criterion applied at 6 and 8 kHz is not age-referenced. Because high-frequency thresholds rise steeply and near-universally with age, an increasing share of what is classified as discordant at older ages is expected to be presbyacusis rather than pathology, and by the eighth decade the great majority of PTA4-normal adults meet the criterion. An age-referenced criterion would be expected to attenuate the observed age gradient substantially, although quantifying it would require a separate analysis referenced to age- and sex-specific normative threshold distributions such as ISO 7029 [26], in which the median 6–8 kHz threshold at older ages lies well above 25 dB HL, and using transducer- and population-appropriate normative data. We retained the fixed 25 dB HL criterion for numerical consistency with the conventional PTA4 threshold and with previous population analyses applying fixed pure-tone cutoffs; it is not an age-normative or clinically validated criterion for 6–8 kHz hearing, and the WHO and GBD frameworks define their thresholds on better-ear PTA4 rather than on any high-frequency average. The older-age estimates should therefore be read as counts of adults exceeding a fixed public-health threshold, not as counts of adults who are abnormal for their age.

## 5. Conclusions

In two independent adult population samples from different countries, better-ear PTA4 classification does not flag a quantifiable and reproducible fraction of adults with elevated high-frequency thresholds (PTA68 > 25 dB HL), including a smaller subgroup with PTA68 > 40 dB HL, nor the majority of those with an interaural PTA68 difference ≥ 15 dB. A 15 dB averaged 6–8 kHz interaural difference is common in the general adult population and frequently not clinically consequential, and this figure should not be read as a measure of clinically relevant asymmetric loss; the clinically consequential pattern is the much smaller unilateral-like group (1.5–1.7%), which a better-ear summary does not represent at all. Within the PTA4-normal denominator the discordance rises most steeply between ages 50 and 69 and reaches its highest levels in the oldest age bands, whereas at the population level it peaks in the 50–69 window because most older adults have already moved into PTA4-defined loss; it is consistent with progressive high-frequency presbyacusis, and is accompanied by a 2.5- to 3.5-fold elevation in self-reported hearing trouble after age and sex adjustment, with the phenotype ordering preserved within every age band examined. The disagreement between the two classifiers is expected on arithmetic grounds; what this study establishes is its magnitude, its age structure, and its reproducibility across two measurement systems, together with a self-report correlate. Because the 25 dB HL criterion is not age-referenced, the discordance observed above age 60 increasingly represents expected high-frequency presbyacusis rather than unexpected pathology. Reporting a high-frequency companion metric (PTA68 or PTA346) alongside PTA4 would make this information visible without displacing the existing PTA4-based classification system.

## Figures and Tables

**Table 2:** Age-stratified prevalence of PTA4-discordant HF loss (PTA68 > 25 dB HL within PTA4-normal adults).

Age Group	HÖRSTAT % (95% CI)	NHANES Unweighted % (95% CI)	NHANES Survey-Weighted % (95% CI)
20–29	1.6 (0.6–4.0)	3.3 (2.5–4.2)	3.4 (2.4–4.9)
30–39	4.1 (2.4–6.9)	6.1 (5.1–7.4)	5.8 (4.5–7.4)
40–49	12.3 (9.8–15.4)	19.3 (17.4–21.4)	21.8 (18.1–26.1)
50–59	34.9 (31.0–39.0)	40.3 (37.8–42.8)	42.2 (37.8–46.8)
60–69	64.9 (60.4–69.2)	60.8 (58.0–63.5)	65.7 (60.9–70.3)
70–79	85.9 (81.3–89.6)	72.9 (68.4–76.9)	76.7 (70.0–82.2)
80+	90.2 (80.2–95.4)	88.3 (79.3–93.7)	86.6 (70.4–94.6)
Overall (PTA4-normal only)	34.3 (32.5–36.2)	26.8 (25.8–27.7)	28.2 (25.9–30.5)
Overall population (all valid)	28.1 (26.5–29.7)	23.6 (22.7–24.5)	24.9 (23.0–26.9)

All age-stratified rows use the within-PTA4-normal denominator (PTA68 > 25 dB HL|PTA4 ≤ 25 dB HL). The final ‘Overall population’ row uses the full valid-bilateral denominator (all adults with valid PTA4 and PTA68) for comparability with published prevalence figures. Wilson CI for unweighted; logit design-adjusted CI for NHANES survey-weighted estimates.

## Data Availability

The German HÖRSTAT/Aalen audiometric data are publicly available in the Dryad Digital Repository (von Gablenz P, Hoffmann E, Holube I. Data from: Gender-specific hearing loss in German adults aged 18 to 84 years compared to US-American and current European studies. Dryad Digital Repository; 2020. doi:10.5061/dryad.cfxpnvx27). The NHANES data are publicly available from the US Centers for Disease Control and Prevention, National Center for Health Statistics (https://www.cdc.gov/nchs/nhanes/, accessed on 28 July 2026). The analysis code is available from the corresponding author upon reasonable request.

## Supplementary Materials

The following supporting information can be downloaded at: https://www.mdpi.com/article/10.3390/audiolres16040111/s1, Table S1: Three estimands of the PTA4–PTA68 classification discordance in PTA4-normal adults; Table S2: Age-stratified PTA68 missingness in NHANES among adults with valid bilateral PTA4, by non-response mechanism; Table S3: Assumption-free (Manski) bounds on the conditional discordance by age band (NHANES, survey-weighted); Table S4: NHANES self-reported hearing trouble (AUQ054 ≥ 3) by audiometric phenotype within age bands (survey-weighted); Table S5: Boundary sensitivity of the PTA4-discordant HF classification to high-frequency measurement error (NHANES, survey-weighted); Table S6: Interaural asymmetry prevalence under alternative cutoffs; STROBE checklist: Reporting checklist of items for cross-sectional studies (STROBE Statement). Reference [27] is cited in the supplementary materials.
