# Supplementary material for: Quantifying What Better-Ear PTA4 Does Not Capture: High-Frequency and Asymmetric Classification Discordance in Two Independent Adult Population Samples from Germany and the United States
Source: Audiol Res. 2026 Jul 31;16(4):111. doi: 10.3390/audiolres16040111 (PMC13510126; doi:10.3390/audiolres16040111)
Supplement: Supplementary file 1 [file audiolres-16-00111-s001.zip › audiolres-4447215-supplementary.pdf]

# Supplementary Materials

*Quantifying what better-ear PTA4 does not capture: high-frequency and asymmetric classification discordance in two independent adult population samples from Germany and the United States*

Supporting tables from the design-based re-analysis (HÖRSTAT/Aalen and pooled NHANES 2011–March 2020). Estimands and bounds are defined in the Methods; all values reproduce the primary analysis.

**Table S1.** Three estimands of the PTA4–PTA68 classification discordance in PTA4-normal adults.

| Estimand | HÖRSTAT | NHANES (survey-weighted) |
|---|---|---|
| 1. Population-level discordance — (PTA4-normal and PTA68 > 25) / all valid adults | 28.1% (26.5–29.7) | 24.9% (23.0–26.9) |
| 2. Conditional discordance (primary) — PTA68 > 25 among PTA4-normal adults | 34.3% (32.5–36.2) | 28.2% (25.9–30.5) |
| 3. Share of HF loss missed — of all PTA68 > 25, % retaining a normal PTA4 | 61.4% (58.8–63.9) | 68.9% (66.4–71.3) |

***Note.*** *HÖRSTAT confidence intervals are Wilson intervals on the unweighted sample (no public weights). NHANES values are survey-weighted with first-order Taylor-linearized confidence intervals (weights, SDMVSTRA strata, SDMVPSU clusters). Estimand 2 is reported as the primary contrast in the main text; estimands 1 and 3 are companions.*

**Table S2.** Age-stratified PTA68 missingness in NHANES among adults with valid bilateral PTA4, by non-response mechanism.

| Age band (yr) | n | PTA68 missing | 6 or 8 kHz "no response" (666) | 8 kHz "could not obtain" (888) |
|---|---|---|---|---|
| 20–29 | 1,694 | 0.0% | 0.0% | 0.0% |
| 30–39 | 1,626 | 0.1% | 0.1% | 0.0% |
| 40–49 | 1,602 | 0.2% | 0.1% | 0.0% |
| 50–59 | 1,615 | 0.2% | 0.1% | 0.1% |
| 60–69 | 1,576 | 1.3% | 1.2% | 0.0% |
| 70–79 | 796 | 14.8% | 14.3% | 0.4% |
| 80+ | 434 | 38.2% | 36.9% | 1.2% |

***Note.*** *Denominator: the 9,343 NHANES adults with valid bilateral PTA4 — the population eligible for the conditional analysis. "PTA68 missing" is the proportion lacking a defined better-ear 6–8 kHz mean. Missingness is negligible below age 70 (≤ 1.3%) and rises to 14.8% (70–79) and 38.2% (80+), driven overwhelmingly by a "no response" code at 6 or 8 kHz (code 666, a profound-loss result) rather than the "could not obtain" code (888) recoded in sensitivity analysis S5; the 888 recode therefore moves estimates negligibly because it addresses the minority code. Of the 315 PTA4-valid adults missing PTA68 (almost all aged 70+), the large majority already meet the PTA4-defined loss criterion and so fall outside the PTA4-normal denominator, which is why the conditional estimate is insensitive to this drop-out (Table S3).*

**Table S3.** Assumption-free (Manski) bounds on the conditional discordance by age band (NHANES, survey-weighted).

| Age band (yr) | Complete-case point | Lower bound | Upper bound | PTA4-normal n | PTA68 missing |
|---|---|---|---|---|---|
| 50–59 | 42.2% | 42.1% | 42.3% | 1,440 | 0.1% |
| 60–69 | 65.7% | 65.4% | 65.9% | 1,204 | 0.3% |
| 70–79 | 76.7% | 73.7% | 77.6% | 430 | 4.0% |
| 80+ | 86.6% | 76.2% | 88.2% | 90 | 14.4% |

***Note.*** *Bounds are obtained by setting every missing PTA68 to its most and least favourable value, requiring no missing-data assumption. The "PTA4-normal n" and PTA68 missingness shown here are within the PTA4-normal subgroup (the conditional denominator); they are lower than the all-PTA4-valid figures in Table S2 because adults who lose PTA68 are disproportionately those with PTA4-defined loss, who are excluded from this denominator. The monotone age gradient holds under every scenario; only the 80+ band (n = 90 PTA4-normal) widens materially. This bound complements the 888 recode reported as S5 in Table 8 of the main text.*

**Table S4. NHANES self-reported hearing trouble (AUQ054 ≥ 3) by audiometric phenotype within age bands (survey-weighted).**

| Age band (yr) | Reference | PTA4-discordant mild | PTA4-discordant strong |
|---|---|---|---|
| 20–39 | 8.4 (7.1–9.8; n = 3,126) | 26.2 (17.0–38.0; n = 123) | 41.5 (25.2–59.8; n = 30) † |
| 40–49 | 10.0 (7.9–12.5; n = 1,257) | 28.1 (20.1–37.8; n = 229) | 50.3 (37.2–63.5; n = 71) |
| 50–59 | 18.0 (13.9–23.1; n = 859) | 30.2 (24.6–36.4; n = 384) | 39.4 (32.1–47.2; n = 195) |
| 60–69 | 13.7 (8.6–21.0; n = 471) | 21.1 (15.7–27.9; n = 383) | 31.2 (25.9–37.1; n = 346) |
| 70+ | 8.4 (4.7–14.8; n = 121) | 14.0 (7.2–25.4; n = 135) † | 24.5 (18.4–31.9; n = 234) |

*Note. Each cell gives the survey-weighted percentage reporting any hearing trouble (AUQ054 ≥ 3), the design-adjusted 95 % confidence interval and the unweighted cell size. Percentages and intervals were computed by the same method as Table 6 of the main text (Taylor linearisation with logit-transformed limits; design df = 57), with the phenotype × age-band cell entered as an estimation domain so that the full survey design is retained. Cell sizes total 7,964, matching Table 6. The ordering of the three phenotypes is preserved in every age band. Consistent with the main text (Section 3.8), the reference and strong intervals are separated in all five bands, whereas the reference and mild intervals overlap in the 60–69 and 70+ bands; the table therefore indicates that age alone does not fully explain the ordering rather than establishing a uniform gradient, and residual confounding within these broad bands cannot be excluded. The proportion reporting trouble within the reference group declines above age 60 because adults who retain a PTA68 at or below 25 dB HL into the eighth decade are a highly selected group with exceptionally well-preserved hearing; the reference group is therefore not comparable in composition across age bands, and the informative comparison is between phenotypes within a band rather than across bands. † Estimate does not meet the NCHS Data Presentation Standards for Proportions (Parker JD et al. Vital Health Stat 2(175). 2017): the 20–39 strong cell has an effective sample size below 30 and a Korn–Graubard interval wider than 30 percentage points, and the 70+ mild cell has a Korn–Graubard relative interval width above 130 % of the estimate. Both are retained rather than suppressed because they carry the ordering information this table exists to show, and both should be read as imprecise.*

**Table S5. Boundary sensitivity of the PTA4-discordant HF classification to high-frequency measurement error (NHANES, survey-weighted).**

| Specification | Result |
|---|---|
| Observed (primary analysis) | 28.2 % |
| PTA68 within ±3 dB of the boundary | 14.5 % of PTA4-normal adults |
| PTA68 within ±5 dB of the boundary | 23.2 % of PTA4-normal adults |
| Added error, per-frequency SD 3 dB | 30.2 % |
| Added error, per-frequency SD 5 dB | 30.8 % |
| Added error, per-frequency SD 7.6 dB | 31.9 % |
| SIMEX extrapolation to zero error (SD 5 dB) | 23.8 % |

*Note. NHANES 6 and 8 kHz retest thresholds (AUXR6K, AUXR8K) exist but are contributed almost entirely by severely impaired ears (median initial threshold 75 dB HL; no boundary crossings among 73 ear-level pairs) and therefore cannot inform misclassification at the 25 dB HL boundary; see Section 3.11. Simulated error is independent Gaussian noise added to each constituent frequency. The 7.6 dB setting is the single-measurement standard deviation implied by the observed 6–8 kHz retest difference standard deviation of 10.8 dB under an assumption of independent, equal-variance repeat measurements (10.8/√2 = 7.6); because errors at 6 and 8 kHz are in part correlated within an ear through transducer placement, it should be read as a conservative upper scenario. These are assumption-based sensitivity scenarios, not empirical estimates of high-frequency test–retest reliability. SIMEX uses lambda = 0, 0.5, 1.0, 1.5 and 2.0 with quadratic extrapolation to lambda = −1.*

**Table S6. Interaural asymmetry prevalence under alternative cutoffs.**

| Asymmetry cutoff | HÖRSTAT PTA4 | HÖRSTAT PTA68 | NHANES PTA4 | NHANES PTA68 |
|---|---|---|---|---|
| ≥ 10 dB | 10.1 % | 31.9 % | 10.7 % | 34.6 % |
| ≥ 15 dB (primary) | 5.3 % | 16.4 % | 5.1 % | 16.6 % |
| ≥ 20 dB | 3.7 % | 8.7 % | 3.2 % | 9.2 % |

*Note. Denominators are participants with valid bilateral values for the metric concerned (HÖRSTAT n = 3,105 for PTA4 and 3,027 for PTA68; NHANES n = 9,343 and 9,035). Unweighted proportions. The 15 dB cutoff is the one used in the main text.*

**STROBE checklist.** Reporting checklist of items for cross-sectional studies (STROBE Statement).

| Item | STROBE recommendation (cross-sectional studies) | Page(s) |
|---|---|---|
| **Title and abstract** | | |
| 1 | (a) Indicate the study's design with a commonly used term in the title or the abstract. (b) Provide in the abstract an informative and balanced summary of what was done and what was found. | 1–2 |
| **Introduction** | | |
| 2 | Background/rationale — explain the scientific background and rationale for the investigation being reported. | 2–3 |
| 3 | Objectives — state specific objectives, including any prespecified hypotheses. | 2, 3 |
| **Methods** | | |
| 4 | Study design — present key elements of study design early in the paper. | 2, 3 |
| 5 | Setting — describe the setting, locations, and relevant dates, including periods of recruitment, exposure, and data collection. | 3–4 |
| 6 | Participants — give the eligibility criteria, and the sources and methods of selection of participants. | 3–4, 8 |
| 7 | Variables — clearly define all outcomes, exposures, predictors, potential confounders, and effect modifiers. Give diagnostic criteria, if applicable. | 3–4 |
| 8 | Data sources/measurement — for each variable of interest, give sources of data and details of methods of assessment (measurement). | 3–4 |
| 9 | Bias — describe any efforts to address potential sources of bias. | 3–5, 21 |
| 10 | Study size — explain how the study size was arrived at. | 8 (Fig. 1) |
| 11 | Quantitative variables — explain how quantitative variables were handled; describe which groupings were chosen and why. | 5, 10 |
| 12 | Statistical methods — (a) all methods, including control for confounding; (b) subgroups and interactions; (c) how missing data were addressed; (d) analytical methods accounting for the sampling strategy; (e) sensitivity analyses. | 3–5 |
| **Results** | | |
| 13 | Participants — (a) numbers at each stage of study; (b) reasons for non-participation at each stage; (c) consider use of a flow diagram. | 8 (Fig. 1) |
| 14 | Descriptive data — (a) characteristics of participants and information on exposures and potential confounders; (b) number of participants with missing data for each variable of interest. | 6–8; Table S2 |
| 15 | Outcome data — report numbers of outcome events or summary measures. | 8–9 |
| 16 | Main results — (a) unadjusted and, where applicable, confounder-adjusted estimates with their precision (e.g., 95% CI); (b) report category boundaries when continuous variables were categorized. | 9–15 |
| 17 | Other analyses — report other analyses done (e.g., subgroups and interactions, sensitivity analyses). | 16–18 |
| **Discussion** | | |

| Item | STROBE recommendation (cross-sectional studies) | Page(s) |
|---|---|---|
| 18 | Key results — summarise key results with reference to study objectives. | 18 |
| 19 | Limitations — discuss limitations, taking into account sources of potential bias or imprecision; discuss both direction and magnitude of any potential bias. | 21 |
| 20 | Interpretation — give a cautious overall interpretation considering objectives, limitations, multiplicity of analyses, results from similar studies, and other relevant evidence. | 18–22 |
| 21 | Generalisability — discuss the generalisability (external validity) of the study results. | 20–22 |
| **Other information** | | |
| 22 | Funding — give the source of funding and the role of the funders for the present study and, if applicable, for the original study on which the present article is based. | 21* |

***Note.*** *Checklist follows the STROBE Statement for cross-sectional studies (von Elm E, et al. Lancet 2007;370:1453–1457). Page numbers refer to the revised clean manuscript. *Item 22: the original cohorts and data sources (HÖRSTAT/Aalen; NHANES) are identified in the Data availability statement (p. 23); the funding and conflict-of-interest statements for the present secondary analysis are completed at submission.*
